# The Relationship Between Face Mask Use and Face-Touching Frequency in Public Areas: Naturalistic Study

**DOI:** 10.2196/43308

**Published:** 2023-05-29

**Authors:** Sydney Niesen, Daniel Ramon, Rhonda Spencer-Hwang, Ryan Sinclair

**Affiliations:** 1 San Diego State University San Diego, CA United States; 2 Loma Linda University Loma Linda, CA United States; 3 Loma Linda University School of Public Health Loma Linda, CA United States

**Keywords:** COVID-19, mask wearing, face-touching, self-inoculation, public health, digital surveillance, webcam video, prevention, health risk, health, risk, mask, surveillance, transmission, behavior

## Abstract

**Background:**

Throughout the COVID-19 pandemic in the United States, a major public health goal has been reducing the spread of the virus, with particular emphasis on reducing transmission from person to person. Frequent face touching can transmit viral particles from one infected person and subsequently infect others in a public area. This raises an important concern about the use of face masks and their relationship with face-touching behaviors. One concern discussed during the pandemic is that wearing a mask, and different types of masks, could increase face touching because there is a need to remove the mask to smoke, drink, eat, etc. To date, there have been few studies that have assessed this relationship between mask wearing and the frequency of face touching relative to face-touching behaviors.

**Objective:**

This study aimed to compare the frequency of face touching in people wearing a mask versus not wearing a mask in high–foot traffic urban outdoor areas. The purpose of this study was to assess if mask wearing was associated with increased face touching.

**Methods:**

Public webcam videos from 4 different cities in New York, New Jersey, Louisiana, and Florida were used to collect data. Face touches were recorded as pedestrians passed under the webcam. Adult pedestrians wearing masks were compared to those not wearing masks. Quantitative measures of frequency, duration, site of touch, and oral activities were recorded. Linear regression analysis was used to assess the association between mask use and face touching.

**Results:**

Of the 490 observed subjects, 241 (49.2%) were wearing a mask properly and 249 (50.8%) were not. In the unmasked group, 33.7% (84/249) were wearing it improperly, covering the mouth only. Face touching occurred in 11.4% (56/490) of the masked group and 17.6% (88/490) in the unmasked group. Of those who touched their face, 61.1% (88/144) of people were not wearing a mask. The most common site of face touching was the perioral region in both groups. Both the masked and unmasked group had a frequency of face touching for 0.03 touches/s. Oral activities such as eating or smoking increased face touching in the unmasked group.

**Conclusions:**

Contrary to expectations, non–mask-wearing subjects touched their face more frequently than those who were wearing a mask. This finding is substantial because wearing a face mask had a negative association with face touching. When wearing a mask, individuals are less likely to be spreading and ingesting viral particles. Therefore, wearing a mask is more effective in preventing the spread of viral particles.

## Introduction

Over the past 2 and a half years, the world has been throttled with a massive pandemic. In August 2020, the COVID-19 disease had caused more than 29,880 deaths in the United States [1]. At some point in time during the pandemic, many county jurisdictions had mandates in place that required face masks to be worn in public outdoor areas to reduce the transmission of respiratory viruses. This recommendation was based on evidence from the outbreak of severe acute respiratory syndrome (SARS) in 2003 followed by the hemagglutinin type 5 neuraminidases type 1 (H5N1) and hemagglutinin type 1 neuraminidases type 1 (H1N1) influenza outbreaks [2]. During these previous outbreaks, it was found that people who are infected with respiratory viruses have the potential to transmit viruses through respiratory secretions that become airborne or adhere to public surfaces [3]. This ultimately justified the mandated use of face masks and the recommendation of other infection prevention practices (eg, frequent handwashing to reduce the spread of respiratory secretions). Along with these recommendations, hand to face contact is another important behavioral factor to control the spread of infectious disease. An exposure assessment is an ideal tool to measure the use of masks, handwashing, and the interaction where face masks impact the frequency of face touching.

Some hypotheses proposed by popular media early in the pandemic claimed that wearing a face mask can heighten facial awareness and sensitivity, prompting an increase in face touching [4]. Such activity can work against the barrier concept of mask wearing. Other concerns addressed a fear of breathing difficulties, constitutional rights being taken away, and hygiene concerns [5]. The idea that an increase in face touching occurs when wearing a mask was introduced for a short time by the US Surgeon General at the beginning of the pandemic [6].

Microbial transport from hand to face has been evaluated in several microbial risk assessments and used to advocate for better handwashing practices in clinical, public, and private environments [7]. Recent studies have investigated face-touching behaviors because they are a known risk for disease transmission. One study suggests that wearing a face mask is associated with decreased face touching, thereby enhancing the protection barrier for which the masks were originally designed [8]. Another study comparing face touching before and during the pandemic found that the frequency of face touching decreased as mask mandates were being implemented [9].

Despite the current published studies, there needs to be an investigation of face-touching behavior at highly frequented outdoor public sites where different activities of human behavior can be naturally observed. For this reason, our study is needed to evaluate how daily human activities affect human behavior and the frequency of face touching. We tested the hypothesis that wearing a face mask will increase face touching while engaged in different activities such as eating, drinking, or smoking.

## Methods

### Study Overview

This study used a video-based, naturalistic, and observational approach to assess the relationship between face mask use and face-touching behaviors of people in public spaces. Public webcams from EarthCam [10] were used to conduct real-time observations in New York, Louisiana, Florida, and New Jersey. Data were recorded on different face-touching behaviors in high-traffic public locations. This study was designed to be empirically focused and methodologically quantitative.

### Recruitment

The target population included people from different ages but were categorized as either adults or children. Those viewed as 16 years of age or older were considered adults, and those younger were classified as children. Individuals were also categorized in the observation as being with or without a mask and whether or not they were observed eating, drinking, or smoking. For individuals to be included, the lighting had to be clear enough to differentiate their hands and face.

EarthCam was used to remotely view and record real-time footage of popular locations in the United States. The locations chosen for this study were selected based on several important factors that included a high density of people, video resolution, and the proximity of the camera to the people to be able to view face masks and behaviors of interest to this study. Five locations were selected based on this criteria: North and South Seaside Heights, New Jersey; Bourbon Street in New Orleans, Louisiana; Times Square in New York City, New York; and Key West, Florida.

### Observation Instrument

**Figure 1:**
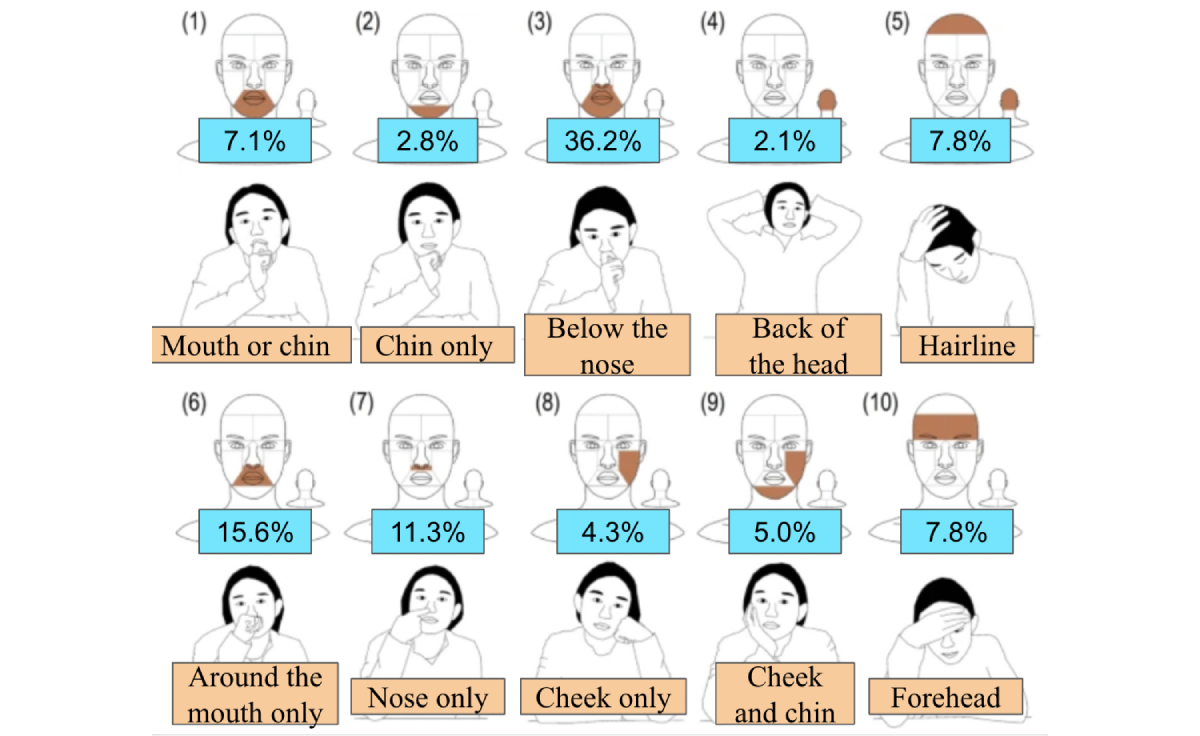
Face touch locations for 10 regions showing the percentage of study participants observed to contact that region. Image adapted from Zhang et al [11], which is published under Creative Commons Attribution 4.0 International License [12].

A survey form using Google Forms was used to structure data entry on subject observations from recorded videos available on EarthCam. These videos were recorded using the screen capture function of a multimedia file player (QuickTime Player, Apple) and then uploaded to a file folder in Google Drive for collaboration. Each file was limited to 3 minutes to conserve file size and allow for systematic review. Recordings were made from October 1, 2020, to November 21, 2020, consistently between 3 and 5 PM (PST) for Tuesdays and Thursdays and between 8 and 10 AM (PST) for Saturdays and Sundays. The structured survey form consisted of observed demographics that included gender appearance, categorized as heteronormative male or female, and age, distinguished as child or adult. The time and date of the recorded observation, the location of the observation, and the duration of the walk was based on when the subject entered and exited the frame. The form also included the number of face touches and the duration of each touch for up to 3 consecutive touches. Each touch was then classified based on where the person touched their face (Figure 1) and if any of those touches were done while eating, drinking, vaping, or smoking. Lastly, the form recorded several types of masks worn and the overall mask-wearing style, classified as correct when it was covering the nose and mouth or incorrect when it covered the nose or mouth only or when worn on the chin or other areas.

### Video Data Review and Record

Observations were made on the web via live public webcams posted on the EarthCam website at various locations. Individuals were selected for inclusion in the study if their hands and face were clearly visible in the frame for at least 5 seconds and not more than 300 seconds. Subjects were included in the observation starting from when they entered the frame to when they exited the frame or when their hands and face became unclear. The video files were then renamed with a predefined format and transferred to a Google Drive account for storage.

Observations were recorded using Google Forms to include time and date, location, age, the duration of the walk, the number of face touches, the duration of each touch up to 3 touches, the part of face touched, oral activity, mask-wearing style, the type of mask, and whether or not they were by themselves or with others. The longest observations were often made in Time Square, New York, because people were observed with a wide field of view sitting in a public courtyard. The shortest observations were in Key West, Florida, due to the placement of the camera by a narrow sidewalk with no benches. Data are available in Multimedia Appendix 1.

### Ethical Considerations

The Loma Linda University institutional review board (IRB) determined that this research does not meet the definitions of human subject research and does not need or require IRB review or approval. The IRB listed 3 reasons. First, it does not obtain or receive private individually identifiable information. Second, there are no data or specimens collected specifically for use in this study. Third, the study does not have direct intervention or interaction with study subjects. The notice of determination from the Loma Linda University IRB was given the number 5210315.

### Statistical Analysis

#### Data Analysis

We used SPSS (version 27; IBM Corp) statistical software to organize data and report frequencies. Within SPSS, we assessed our hypothesis questions by evaluating the asymptotic significance from Pearson chi-square test and by evaluating the unstandardized β with its asymptotic significance using multiple linear regression, seen in Table 1.

To determine the ideal sample size, we used R statistical software with the *pwr* package (R Foundation for Statistical Computing) [13] to analyze the minimum observations that would detect mask wearing with a medium effect size of 0.5, a significance level of .01, and a power of 90%. To observe the difference in mask-wearing practices, we needed 350 participants for this power and sample size. We aimed to collect beyond that number to allow a buffer for anticipated stratification in bivariate and regression analyses.

**Table 1:** Multiple linear regression for the frequency of face touching in relation to the type of face mask among mask wearers (n=434)^a^.

Linear regression model	Unstandardized β	Coefficients SE	Standardized coefficients β	*t* test (*df=1*)	*P* value
Frequency of touching face (constant)	0.38	0.266	N/A^b^	1.428	.15
Washable mask	0.132	0.236	0.029	0.558	.58
Surgical mask	–0.192	0.273	–0.036	–0.702	.48
N95 mask	–0.374	0.74	–0.023	–0.506	.61
Neck gaiter	–0.3	0.646	–0.021	–0.464	.64
Other type of mask	–0.516	1.535	–0.015	–0.336	.74
Oral activity	1.251	0.376	0.157	3.325	.001
With others or by themselves (no or yes)	0.273	0.257	0.05	1.06	.29
Gender	–0.207	0.207	–0.047	–0.999	.32

^a^Dependent variable: the frequency of face touches with “zero face touching” as the reference group.

^b^N/A: not applicable.

#### Data Exclusion

Individuals where the sun or a streetlight washed out their face or hands were excluded. Additionally, if the individual was in the frame for less than 5 seconds, they were excluded because it was considered an insufficient amount of time for this study.

## Results

### Selected Population

We selected 490 individuals from August to November 2020 who met the study criteria. Over 65 hours of video were reviewed involving subject observations at 4 different United States locations including New York, New York (n=283, 57.8%); Seaside Heights, New Jersey (n=14, 2.9%); Key West, Florida (n=18, 3.7%); and New Orleans, Louisiana (n=175, 35.7%). Table 2 represents the demographics of all the subjects involved in the study with a total of 274 (55.9%) male and 216 (44.1%) female subject observations. Of these, we observed 20 (4.1%) individuals that were identified as children and appeared to be younger than 16 years old. For sample size, we needed a minimum of 241 observations for the detection of mask wearing with a medium effect size of 0.5, a significance level of .01, and a power of 90%. We collected 490 observations to allow a buffer for stratification in bivariate and regression analyses.

Most observations were made at Bourbon Street and Times Square due to the high foot traffic in those cities. The 3 other cameras (2 in Seaside Heights and 1 in Key West) had frequent visual obstructions from direct sunlight or fog, were too far away from individuals, or had a small field of view with several individuals that passed too quickly.

**Table 2:** Demographic characteristics of people observed in New Orleans, New York, Florida, and New Jersey.

Characteristic		Mask (n=241)	No mask (n=249)	Total (n=490)	Chi-square test					
					Chi-square, (*df*)		Value, n		*P* value	
**Gender appearance, n (%)**						0.0026 (1)		488		.96
	Male	120 (49.8)	154 (61.8)	274 (55.9)						
	Female	121 (50.2)	95 (38.2)	216 (44.1)						
**Age, n (%)**						0.2725 (1)		489		.60
	Child	11 (4.6)	9 (3.6)	20 (4.1)						
	Adult	230 (95.4)	240 (96.4)	470 (95.9)						
**Face touch, n (%)**						8.64 (1)		490		<.05
	Yes	56 (23.2)	88 (35.3)	144 (29.4)						
	No	185 (76.8)	161 (64.7)	346 (70.6)						
**Multiple face touch, n (%)**						9.672 (1)		490		<.05
	Yes	17 (7.1)	40 (16.1)	57 (11.6)						
	No	224 (92.9)	209 (83.9)	433 (88.4)						
**Long face touch, n (%)**						5.864 (1)		490		<.05
	Yes	29 (12)	50 (20)	79 (16.1)						
	No	212 (88)	199 (80)	411 (83.9)						
**Site of face touch, n**						15.68 (9)		144		<.05
	1	5	10	15						
	2	1	3	4						
	3	21	28	49						
	4	1	2	3						
	5	4	5	9						
	6	2	18	20						
	7	9	7	16						
	8	5	2	7						
	9	1	6	7						
	10	7	7	14						
**Oral activity, n (%)**						36.84 (1)		490		<.05
	Yes	1 (0.4)	38 (15.3)	39 (8)						
	No	240 (99.6)	211 (84.7)	451 (92)						
**Oral detail, n (%)**						19.11 (3)		484		<.05
	Smoking	0 (0)	11 (4.4)	11 (2.2)						
	Vaping	0 (0)	2 (0.8)	2 (0.4)						
	Drinking	1 (0.4)	19 (7.6)	20 (4.1)						
	Eating	0 (0)	6 (2.4)	6 (1.2)						
Frequency of face touch (touch/s)		0.03	0.03	0.03						
**Location, n (%)**						19.11 (3)		484		<.05
	New Orleans	70 (29)	105 (42.2)	175 (35.7)						
	New York	161 (66.8)	122 (49)	283 (57.8)						
	New Jersey	6 (2.5)	8 (3.2)	14 (2.9)						
	Florida	4 (1.7)	14 (5.6)	18 (3.7)						

### Face-Touching Observations

From our observations, the majority of the population touched their face in the same area. As seen in Figure 1, area 3, below the nose to the bottom of their chin, was the most common place that subjects touched their face. Most other areas fell within this larger area and were coded as areas 1, 2, 3, 6, or 7 (mask region as “mskreg1”). Area 3 was the most frequently observed, because in many cases, the video quality was insufficient to determine the exact location.

We observed a total of 144 people touching their face at least once, with many touching different regions of their face. From the population that touched their face, 88 (61.1%) people were not wearing a mask and 56 (38.9%) people were wearing a mask. We counted a total of 273 discrete face touches in all 490 observed subjects. Of everyone who touched their face for more than one second, 37% (29/79) were wearing a mask and 63% (50/79) were not wearing a mask. Face touches longer than 6 seconds accounted for 4.2% (6/144) of all face touch observations, with a 15-second touch being the lengthiest touch (n=1).

Of those who were wearing a mask, only 7.1% (17/241) touched the face more than once, seen on Table 3. Of those who touched their face for longer than 1 second, 37% (29/79) touched their face more than once.

**Table 3:** Frequency of single touch versus multiple touches shown across subject’s oral activity, touch duration and mask wearing.

Variable	Multitouch	No multitouch	Chi-square test		
			Chi-square (*df*)	Value, n	*P* value
Oral activity (n=39)	16 (41)	23 (59)	35.6 (1)	490	<.001
Long touch duration (>1 second; n=79)	29 (36.7)	50 (63.3)	57.6 (1)	490	<.001
Wearing a mask (n=241)	17 (7.1)	224 (92.9)	9.67 (1)	490	.002

### Mask-Wearing Observations

Mask-wearing style was recorded to observe if subjects were wearing their mask properly, covering their nose and mouth. Subjects who were only covering their mouth with their mask, wearing it as a chin strap, or taking their mask on and off were considered to be not wearing a mask for this study. In Table 4, the frequency of mask style is presented.

Washable masks (homemade or manufactured) accounted for the most frequently observed type of mask at 53.1% (128/241). At 43.2% (104/241), disposable surgical masks were the second most frequently observed type of mask. Subsequent observations of each additional type of mask drastically fell to less than 8%: N95 masks only accounted for 3.7% (9/241) of all observed face masks. Mask types were only recorded if the subject was wearing a mask properly.

**Table 4:** Frequency of mask style.

Mask styles	Frequency observed (N=490), n (%)
Covering nose and mouth	241 (49.2)
Not wearing one	158 (32.2)
Covering mouth only	24 (4.9)
Chin strap	49 (10)
Partially wearing mask^a^	18 (3.7)

^a^Partially wearing mask consists of taking the mask on and off and dangling it from the ear.

## Discussion

### Principal Findings

This exposure assessment used a naturalistic observation method and found that the average face touching frequency was 0.03 touches per second or 1.8 touches per minute for over 400 individuals. This rate was comparable to other studies, which found a frequency of 0.8 touches per minute in an indoor environment [7] for 10 subjects. Our original hypothesis was that wearing a face mask will increase the frequency of face touching. Contrary to our hypothesis, the regression shows a negative association between mask wearing and the frequency of face touching.

The results indicate that almost half of the observations made (241/490, 49.2%) were of people wearing a mask properly, covering the nose and mouth. In both the mask-wearing and non–mask-wearing groups, the most frequent area of face touch was the space between the nostrils and the chin, as shown in Figure 1. From our observations of individuals that touched their face, 61.1% (88/144) were not wearing a mask. Of all the individuals that touched their face more than once, 70.2% (40/57) of them were not wearing a mask. These observations show that those wearing a mask had a lower face-touching frequency compared to those who were not wearing a mask.

Face masks covering the nose and mouth have been proven to limit the spread of the COVID-19 disease [2,14]. In public spaces, people infected with COVID-19 can contaminate their environment, which will later on contaminate the hands of the general public. Hand to face transmission is a critical transmission route to study in public areas where there are significantly more objects or materials that are likely to carry infection [7]. Face-touching behaviors are important to study as it relates to exposure assessment science. It is crucial to understand how these behaviors impact the spread of disease or viral particles.

### Limitations

The use of public webcam footage only allowed the subjects to be observed for a short walking distance in the few seconds they were in frame of the shot. This is due to the constraints of the focal length in the camera lens. Therefore, subjects were only observed for anywhere between our minimum inclusion criteria of 5 seconds up through the longest recorded duration of 5 minutes. A wider camera lens would have been useful for monitoring subjects at a greater distance and for a longer period of time. However, the constraint of public webcams created a standardized focal length that allowed for a consistent review of the footage. In other similar studies, the monitoring distance and observation time are not clear [15]. Additionally, this study took place exclusively in outdoor public spaces and not in enclosed spaces such as offices, markets, restaurants, etc. Thus, the findings in this study can only be applicable to face-touching behaviors in public spaces and not in enclosed spaces.

### Comparison With Prior Work

The findings of this study concluded that mask wearing is not associated with an increased frequency of face touching. Another study investigated this hypothesis by comparing face-touching behaviors before and during the COVID-19 pandemic [9]. Their study took place in China, Japan, South Korea, and Western Europe and found that the frequency of face touching decreased as mask mandates were being implemented. This is important because it demonstrates that mask wearers have been shown to reduce face-touching behaviors. Therefore, face masks offer a double advantage in decreasing viral transmission through the protection of the oropharyngeal area and decreasing the potential for face-touching frequency.

## Appendix

Multimedia Appendix 1 Video data review and record.

## Abbreviations

IRB: institutional review board
H1N1: hemagglutinin type 1 neuraminidases type 1
H5N1: hemagglutinin type 5 neuraminidases type 1
SARS: severe acute respiratory syndrome
